# Correlation of CS and OCTA biomarkers in CSCR

**DOI:** 10.22336/rjo.2025.86

**Published:** 2025

**Authors:** Shaffi Chopra, Satya Prakash Singh, Gyanmani Pandey, Kamal Jeet Singh, Vinod Kumar Singh, Parul Singh

**Affiliations:** 1 Department of Ophthalmology, M.L.N. Medical College, Prayagraj, Uttar Pradesh, India; 2 Department of Ophthalmology, G.S.V.M. Medical College, Kanpur, Uttar Pradesh, India

**Keywords:** central serous chorioretinopathy, OCTA biomarkers, contrast sensitivity, choriocapillaris, visual quality, BCVA = Best Corrected Visual Acuity, CC = Choriocapillaris, CNV = Choroidal Neovascularization, CS = Contrast Sensitivity, CSCR = Central Serous Chorioretinopathy, CSF = Contrast Sensitivity Function, DCP = Deep Capillary Plexus, FA = Fluorescein Angiography, ICGA = Indocyanine Green Angiography, OCT = Optical Coherence Tomography, OCTA = Optical Coherence Tomography Angiography, PED = Pigment Epithelial Detachment, RPE = Retinal Pigment Epithelium, SD-OCT = Spectral Domain Optical Coherence Tomography, SRD = Serous Retinal Detachment, SRF = Subretinal Fluid, SCP = Superficial Capillary Plexus, SSADA = Split-Spectrum Amplitude Decorrelation Angiography

## Abstract

**Objective (Aim):**

To identify Optical Coherence Tomography Angiography (OCTA) biomarkers in eyes with acute Central Serous Chorioretinopathy (CSCR) and assess their correlation with contrast sensitivity (CS).

**Methods:**

In this prospective observational study, 32 eyes of 30 patients with acute CSCR underwent OCTA, spectral-domain OCT, and fluorescein angiography. Contrast sensitivity was measured using the Pelli-Robson chart.

**Results:**

OCTA revealed three types of abnormalities: dark areas in all eyes, dark spots in five eyes (associated with serous retinal detachment and pigment epithelium detachment), and abnormal vessels in twelve eyes. Mean baseline CS was 1.42, which improved to 1.78 at six-month follow-up (p < 0.001).

**Discussion:**

Although OCTA abnormalities varied, the presence of persistent dark areas correlated with reduced contrast sensitivity, emphasizing that structural recovery does not equate to functional recovery.

**Conclusions:**

Although visual acuity normalized in most cases, persistent OCTA abnormalities and reduced CS highlighted ongoing visual quality impairment, indicating that anatomical recovery does not equate to full functional recovery.

## Introduction

Central serous chorioretinopathy (CSCR) is a prevalent vision-threatening macular disorder, ranked after age-related macular degeneration, diabetic retinopathy, and retinal vein occlusion. It is characterized by a localized serous detachment of the neurosensory retina, often accompanied by focal detachment of the retinal pigment epithelium (RPE) [**1**].

The condition predominantly affects young males but may occur in both sexes. Risk factors include elevated endogenous or exogenous corticosteroids, stress-prone personality traits, pregnancy, smoking, uncontrolled systemic hypertension, certain antibiotics, organ or bone marrow transplantation, and upper respiratory tract infections, including Helicobacter pylori infection [**2-5**].

The typical age of onset ranges from 20 to 50 years [**6**].

The clinical presentation varies depending on the duration and severity of retinal and RPE changes. While acute CSCR usually resolves spontaneously within a few months, chronic or recurrent forms may persist with subretinal fluid (SRF) for more than four months. In the chronic atrophic form, which persists beyond six months, widespread RPE atrophy may be observed with or without fluid retention [**7,8**].

The pathogenesis of CSCR remains incompletely understood. Proposed mechanisms include diffuse RPE dysfunction leading to impaired subretinal fluid absorption, and increased choroidal vascular permeability resulting in elevated hydrostatic pressure [**1,4,9**].

Diagnosis is primarily clinical and supported by fluorescein angiography. Recent advances have introduced optical coherence tomography angiography (OCTA) as a non-invasive imaging modality, enabling detailed visualization and monitoring of the disease [**10-12**].

OCTA functions by detecting motion contrast from erythrocytes within retinal and choroidal vessels, using decorrelation signal processing from repeated B-scans at the same location. This allows three-dimensional visualization of microvasculature without the need for dye injection, and segmentation of individual vascular layers unaffected by leakage or staining [**13-15**].

Previous studies utilizing OCTA have reported varying patterns of choriocapillaris (CC) flow alterations in CSCR [**13-17**].

However, none have explored the correlation between these OCTA findings and contrast sensitivity (CS), an essential subjective visual parameter [**18**].

This prospective observational study aims to investigate OCTA biomarkers in eyes with CSCR and correlate these findings with the clinical course and contrast sensitivity outcomes.

## Materials and methods

### Study Design and Participants

This prospective, single-center, observational study was conducted at a tertiary care eye hospital from December 2021 to December 2022. Patients with acute central serous chorioretinopathy (CSCR), defined by visual symptoms lasting less than three months, were included. Only newly diagnosed, treatment-naïve cases were enrolled. Patients with prior CSCR episodes or coexisting ocular diseases were excluded.

### Ophthalmic Examination

All patients underwent complete ophthalmic evaluation, including best-corrected visual acuity (BCVA) using the Snellen chart, contrast sensitivity with the Pelli-Robson chart, slit-lamp biomicroscopy, and dilated fundus examination. Imaging included fluorescein angiography, spectral-domain optical coherence tomography (SD-OCT), and optical coherence tomography angiography (OCTA).

### OCTA Acquisition

OCTA was performed using the TOPCON 3D OCT-1 Maestro2 system, employing Split-Spectrum Amplitude Decorrelation Angiography (SSADA) to acquire 3 × 3 mm scans centered on the macula (304 × 304 A-scans/volume; acquisition time: 2.6 seconds). Automated segmentation of the superficial capillary plexus (SCP), deep capillary plexus (DCP), outer retina, and choriocapillaris (CC) was performed, with manual corrections where necessary. Structural co-registration was used to confirm OCTA accuracy.

### Quantitative and Qualitative Analysis

Biomarkers, including dark areas, dark spots, and abnormal vessels, were evaluated at SCP, DCP, the outer retina, and the CC levels. These were correlated with central macular thickness (CMT), BCVA, and contrast sensitivity. A follow-up was conducted at 6 months.

### Statistical analysis

Data analysis was performed using SPSS version 23.0 and Microsoft Excel. Continuous variables (e.g., BCVA, contrast sensitivity) were analyzed using paired t-tests; categorical variables were analyzed using Chi-square tests. A p-value < 0.05 was considered statistically significant.

### Ethical Considerations

The study was approved by the Institutional Ethics Committee and complied with the Declaration of Helsinki. Written informed consent was obtained from all participants. Sex- and race/ethnicity-specific data were collected where applicable. No animal experiments were involved. No identifiable patient information has been disclosed.

## Results

Thirty-two eyes of 30 consecutive patients (28 males and two females; mean age: 31.10 ± 5.95 years) diagnosed with central serous chorioretinopathy (CSCR) were included in the study. The mean best-corrected visual acuity (BCVA) was 0.451 ± 0.25 logMAR (range: 0–1.0 logMAR, equivalent to 20/20 to 20/200). Unilateral involvement was observed in 87.5% of eyes, and bilateral involvement in 12.5%. Patients were categorized into two groups based on the duration of subretinal fluid (SRF) resolution: the early resolution group (≤3 months, 16 cases) and the delayed resolution group (>3 months, 11 cases), as shown in **Table 1**. One patient had bilateral persistent disease (2 eyes), and three cases were identified as recurrent CSCR.

**Table 1:** Comparative outcomes between early and delayed SRF resolution groups

Parameter	Early Resolution (≤3 months)	Delayed Resolution (>3 months)	p-value
Mean CMT (μm)	355.12 ± 40.50	412.65 ± 52.30	0.002
Mean CS	1.81 ± 0.16	1.66 ± 0.17	0.014
BCVA (logMAR)	0.21 ± 0.08	0.38 ± 0.15	0.030
Recurrence (%)	0%	27.2%	0.045

At the outer retina level, OCTA revealed abnormal flow in 5 eyes (15.6%). In the remaining 27 eyes (84.3%), no remarkable findings were detected, likely due to projection artifacts from superficial and deep capillary plexuses.

In the choriocapillaris (CC) layer, OCTA segmentation (30–60 μm beneath the RPE) demonstrated three distinct findings: dark areas, dark spots, and abnormal choroidal vessels. Dark areas presented as diffuse or focal, foggy, ill-defined, low-flow regions. Dark spots appeared as distinct, well-defined black zones lacking flow, either isolated or associated with dark areas. Abnormal choroidal vessels were characterized by high-flow, tangled vascular patterns, and abnormal dilation. A strong association between dark areas and SRF was observed in 25 of 32 eyes. Morphological correlations of these OCTA features with corresponding OCT findings are detailed in **Table 2**.

**Table 2:** Correlation of OCTA biomarkers with OCT findings

OCTA Biomarker	Associated OCT Findings	Number of Eyes (n=32)
Dark areas	Subretinal detachment (SRD)	25
Dark spots	Pigment epithelium detachment (PED)	5
Abnormal vessels	Dilated choroidal vessels	12

**Fig. 1:**
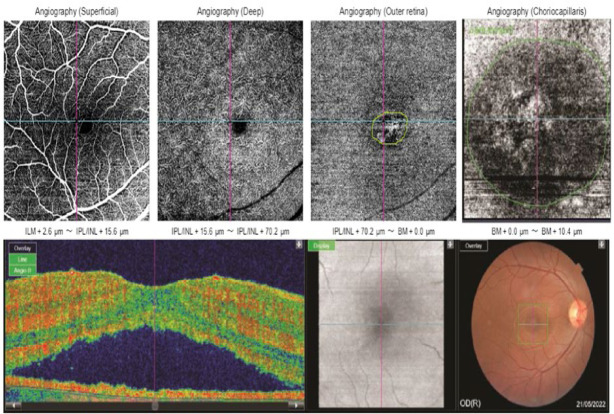
Acute central serous chorioretinopathy in a 32-year-old man. Correlation between optical coherence tomography (OCT) and OCT angiography (OCT). OCTA at the level of the outer retina showing abnormal vessel dilatation, delineated in a green circle. OCTA at the choriocapillaris: the green circle delineates an area of apparent blood flow hypoperfusion and hyperperfusion, called a dark area in this study, measuring 5.84 mm^2^ at the first visit

**Fig. 2:**
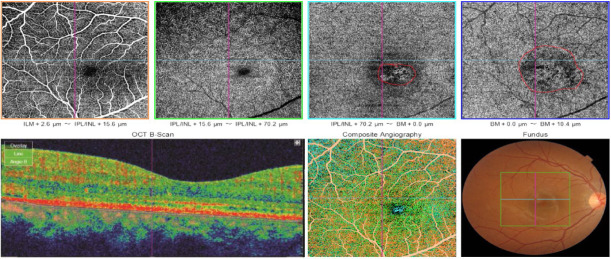
Correlation between optical coherence tomography (OCT) and OCT angiography (OCTA). OCTA at the level of the outer retina showing persistence of abnormal vessel dilatation, delineated in a red circle. OCTA of the choriocapillaris: the red circle delineates an area of apparent hypoperfusion and hyperperfusion, called a dark area in this study, measuring 1.84 mm^2^ at 6 months

Quantitative assessment of OCTA biomarkers and their relationship with contrast sensitivity (CS) is summarized in **Table 3**. All parameters showed statistically significant differences, except for the area of dark spots and the presence of pigment epithelium detachment (PED). The mean circumference of the dark area decreased from 3.94 at baseline to 1.03 at six-month follow-up. The mean central macular thickness (CMT) decreased from 389.72 ± 47.70 μm at baseline to 237.31 ± 41.22 μm after six months. **Fig. 1** and **2** illustrate the changes in OCTA biomarkers at baseline and at the six-month follow-up, respectively.

**Table 3:** Quantitative assessment of OCTA biomarkers and visual function

Parameter	Baseline	6-month Follow-up	p-value
Dark area circumference (mm)	3.94	1.03	<0.001
Central macular thickness (μm)	389.72 ± 47.70	237.31 ± 41.22	<0.001
Contrast sensitivity (CS)	1.42 ± 0.28	1.78 ± 0.17	<0.001

Visual acuity improved significantly, associated with a reduction in CMT, as shown in **Table 4**. Sixteen eyes regained a BCVA of 6/6 by the third month. However, persistent SRF was observed in 2 eyes, and recurrence occurred in 3 eyes. At baseline, patients demonstrated moderate contrast sensitivity loss (mean CS: 1.42 ± 0.28), which changed to 1.47 ± 0.28 at four weeks (not statistically significant). At 24 weeks, the mean CS improved significantly to 1.78 ± 0.17 (p < 0.001). Despite restoration of 6/6 visual acuity in 25 eyes, complete normalization of contrast sensitivity and disappearance of OCTA dark areas were observed in only seven eyes. In the remaining eyes, OCTA biomarkers persisted even after anatomical resolution of SRF, as shown in **Table 5**.

**Table 4:** Distribution of visual and OCTA outcomes at follow-up

Visual Outcome	Number of Eyes
BCVA 6/6 at 3 months	16
Persistent SRF	2
Recurrent CSCR	3
Complete CS + OCTA recovery	7

**Table 5:** Correlation of persistent OCTA biomarkers (dark area) with visual outcomes

Group	Mean CS at 6 months	Eyes with BCVA 6/6	Eyes with Normal CS
Dark Area Resolved (n=7)	1.91 ± 0.07	7 (100%)	7 (100%)
Dark Area Persistent (n=25)	1.72 ± 0.14	18 (72%)	0 (0%)

## Discussion

In this prospective study, we evaluated the clinical and imaging features of acute central serous chorioretinopathy (CSCR) using optical coherence tomography angiography (OCTA) to identify predictive biomarkers and correlate them with visual parameters, such as contrast sensitivity (CS). OCTA is a non-invasive modality for assessing microvascular abnormalities in the outer retina and choriocapillaris (CC), providing insight into disease pathogenesis and prognosis.

Our study involved 32 eyes of 30 patients, with a mean age of 31.10 ± 5.95 years, consistent with previous reports by Liew G et al. [**3**], which documented CSCR prevalence among individuals aged 20–50 years. The disease was predominantly observed in males (93.3%), with a mean age of 31.64 ± 5.77 years. Quantitative evaluation using OCT revealed central macular thickness (CMT) of 301–400 μm in nearly half the cases, with only 5% of eyes showing pigment epithelium detachment (PED), suggesting that while PED can be associated with CSCR, it is not a defining feature in most acute cases.

OCTA enabled the visualization of the vascular architecture at various retinal and choroidal levels, notably at the automatically segmented outer retina and CC. In normal eyes, a consistent CC vascular pattern was observed; however, in CSCR eyes, three key OCTA biomarkers were identified: dark areas, dark spots, and abnormal choroidal vessels. Dark areas were primarily associated with serous retinal detachment (SRD), while dark spots correlated with PED. These flow voids likely represent regions of CC atrophy or hypoperfusion secondary to choroidal congestion, as previously suggested by Yang et al. [**12**] and Costanzo et al. [**14**].

The literature indicates that OCTA is effective not only in detecting CSCR-associated microvascular changes but also in identifying secondary choroidal neovascularization (CNV), especially in chronic cases. In our study, CNV was detected in 5 patients, reinforcing the utility of OCTA for both diagnostic and prognostic purposes. These findings are consistent with previous reports citing OCTA’s high sensitivity for CNV detection.

Contrast sensitivity loss was noted in the majority of patients at baseline and showed only partial improvement over time. Prasad et al. [**18**] observed that CS and color vision may remain impaired despite normalization of visual acuity, particularly in the early resolution group. Similarly, in our study, 25 patients showed persistent dark areas on OCTA, and only 7 achieved complete recovery of CS, despite anatomical resolution of SRF and restoration of 6/6 BCVA in 25 eyes.

Persistent OCTA biomarkers—specifically dark areas—were associated with reduced foveal perfusion, potentially damaging foveal cones, and impacting CS. Although visual acuity may return to normal, approximately 63.6% of patients still reported visual discomfort and dissatisfaction, highlighting the inadequacy of BCVA alone in assessing functional vision outcomes in CSCR.

This study underscores the relevance of OCTA as a follow-up tool in CSCR management. Unlike fluorescein angiography, OCTA provides non-invasive, repeatable, layer-specific imaging that is particularly beneficial for monitoring disease progression. Our findings support a significant correlation between CS and OCTA biomarkers, particularly the persistence of dark areas, which adversely impact visual quality even after apparent anatomical recovery.

## Conclusion

In conclusion, the persistence of hypoperfusion patterns on OCTA and incomplete CS recovery despite restored BCVA suggest that OCTA biomarkers are essential prognostic indicators. Routine OCTA evaluation may aid in comprehensive visual assessment and tailored follow-up of CSCR patients.

## References

[1] Daruich A, Matet A, Dirani A, Bousquet E, Zhao M, Farman N (2015). Mechanisms of disease: central serous chorioretinopathy. Prog Retin Eye Res.

[2] Conrad R, Geiser F, Schilling G, Imbierowicz K, Liedtke R (2000). Central serous chorioretinopathy and psychological distress: associations with Type‑A personality traits and stress coping. Nordic J Psychiatry.

[3] Liew G, Quin G, Gillies M, Fraser‑Bell S (2013). Central serous chorioretinopathy: a review of epidemiology and pathophysiology. Clin Exp Ophthalmol.

[4] Gemenetzi M, De Salvo G, Lotery AJ (2010). Central serous chorioretinopathy: an update on pathogenesis and treatment. Eye (Lond).

[5] Liang ZQ, Huang LZ, Qu JF, Zhao MW (2018). Association between endogenous cortisol level and the risk of central serous chorioretinopathy: a Meta-analysis. Int J Ophthalmol.

[6] Nakatsuka AS, Nazari Khanamiri H, Lam QN, El-Annan J (2013). Intranasal corticosteroids and central serous chorioretinopathy: a case report and literature review. Middle East Afr J Ophthalmol.

[7] Kitzmann AS, Pulido JS, Diehl NN, Hodge DO, Burke JP (2008). The incidence of central serous chorioretinopathy in Olmsted County, Minnesota, 1980-2002. Ophthalmology.

[8] Wang M, Munch IC, Hasler PW, Prünte C, Larsen M (2008). Central serous chorioretinopathy. Acta Ophthalmol.

[9] Casella AMB, Berbel RF, Bressanim GL, Malaguido MR, Cardillo JA (2012). Helicobacter pylori as a potential target for the treatment of central serous chorioretinopathy. Clinics (Sao Paulo).

[10] Eom Y, Oh J, Kim SW, Huh K (2012). Systemic factors associated with central serous chorioretinopathy in Koreans. Korean J Ophthalmol.

[11] Errera MH, Kohly RP, da Cruz L (2013). Pregnancy associated retinal diseases and their management. Surv Ophthalmol.

[12] Yang L, Jonas JB, Wei W (2013). Optical coherence tomography-assisted enhanced depth imaging of central serous chorioretinopathy. Invest Ophthalmol Vis Sci.

[13] Quaranta-El Maftouhi M, El Maftouhi A, Eandi CM (2015). Chronic central serous chorioretinopathy imaged by optical coherence tomography angiography. Am J Ophthalmol.

[14] Costanzo E, Cohen SY, Miere A, Querques G, Capuano V, Semoun O, El Ameen A, Oubraham H, Souied EH (2015). Optical Coherence Tomography Angiography in Central Serous Chorioretinopathy. J Ophthalmol.

[15] de Carlo TE, Romano A, Waheed NK, Duker JS (2015). A review of optical coherence tomography angiography (OCTA). Int J Retina Vitreous.

[16] Feucht N, Maier M, Lohmann CP, Reznicek L (2016). OCT angiography findings in acute central serous chorioretinopathy. Ophthalmic Surg Lasers Imaging Retina.

[17] McClintic SM, Jia Y, Huang D, Bailey ST (2015). Optical coherence tomographic angiography of choroidal neovascularization associated with central serous chorioretinopathy. JAMA Ophthalmol.

[18] Prasad GV, Divya P (2019). Prospective study of role of contrast sensitivity and colour vision in central serous chorioretinopathy. Int J Adv Res.

